# Synthesis and Characterization of Statistical and Block Copolymers of n-Hexyl Isocyanate and 3-(Triethoxysilyl) Propyl Isocyanate via Coordination Polymerization

**DOI:** 10.3390/polym15204113

**Published:** 2023-10-17

**Authors:** Maria Panteli, Dimitra Mantzara, Aikaterini Katara, Ioannis Choinopoulos, Marinos Pitsikalis

**Affiliations:** Industrial Chemistry Laboratory, Department of Chemistry, National and Kapodistrian University of Athens, Panepistimiopolis Zografou, 15771 Athens, Greece; mariap9655@gmail.com (M.P.); di.mitroula1997@hotmail.com (D.M.); katerinakatara01@gmail.com (A.K.)

**Keywords:** n-hexyl isocyanate, 3-(triethoxysilyl) propyl isocyanate, coordination polymerization, half-titanocene complex, statistical copolymers, block copolymers, thermal decomposition

## Abstract

Well-defined statistical copolymers of n-hexyl isocyanate, HIC, and 3-(triethoxysilyl)propyl isocyanate, TESPI, were synthesized via coordination polymerization mechanism, employing a chiral half-titanocene complex as initiator. The monomer reactivity ratios of the statistical copolymers were calculated using linear graphical methods and the computer program COPOINT in the frame of the terminal model. The molecular and structural characteristics of the copolymers were also calculated. The kinetics of the thermal decomposition of the statistical copolymers was studied by Thermogravimetric Analysis, TGA, and Differential Thermogravimetry, DTG, and the activation energy of this process was calculated employing several theoretical models. In addition, block copolymers constituted from PHIC and PTESPI blocks were synthesized by sequential coordination polymerization. All samples were characterized by nuclear magnetic resonance, NMR, spectroscopy and size exclusion chromatography, SEC. The thermal stability of the blocks was also studied by TGA and DTG and compared to the corresponding statistical copolymers.

## 1. Introduction

Poly(alkyl isocyanate)s, PICs, constitute an interesting class of polymeric materials since they adopt helical conformation both in solution and in bulk [1,2,3,4]. It has been shown, employing various characterization techniques, that PICs are stiff-chain polymers. Their properties depend on several parameters, such as temperature, solvent, nature of the isocyanate’s side group and polymer’s molecular weight [1,2,5,6,7,8]. Therefore, they may behave either as rigid rods or as semiflexible worm-like chains. Combination of PICs with other rigid or more flexible chains in diblock copolymers or more complex macromolecular architectures opens new horizons in nanotechnology focusing on the microphase separation, the ordering kinetics and the self-assembly behavior in solution [9,10,11]. Consequently, novel applications are expected to emerge, making these materials possible candidates as optical switches, recognition devices, etc. [12,13,14,15,16].

The synthesis of well-defined structures based on PICs was, for many years, hindered by the lack of efficient synthetic routes to control their molecular characteristics [17,18]. Anionic polymerization based on NaCN initiation was originally reported by Shashoua et al. [5,7]. However, this system, along with other anionic polymerizations utilizing classical initiators, failed to give polymers with predetermined molecular and structural characteristics due to the presence of back-biting reactions leading to the formation of trimers [19].

Two different approaches were then developed to resolve the synthetic drawbacks concerning the polymerization of alkyl isocyanates, ICs. The successful anionic polymerization of ICs was achieved using sodium naphthalenide (Na-Naph) in combination with either the crown ether 15C5 or the salt NaBPh_4_ [9,10,11,12,13,14,15,16,17,18,19,20,21,22]. By this method, a number of homopolymers of ICs (n-octyl, n-hexyl and 3-(triethoxysilyl)propyl isocyanates), as well as the triblock copolymer poly [3-(triethoxysilyl)propyl isocyanate–*b*-styrene-*b*-3-(triethoxysilyl) propyl isocyanate], were synthesized [20,22,23,24]. Well-defined triblock copolymers and pentablock terpolymers of n-hexyl isocyanate (H), styrene (S) and isoprene (I) were synthesized as well [25]. Polymerization with NaBPh_4_ as an additive was found to be more promising than with 15C5 because NaBPh_4_ stabilizes the amidate anion of the growing polyisocyanate (common ion effect) by forming a tight ion pair, which prevents the back-biting reaction. Furthermore, the back-biting reaction is inhibited by the very bulky tetraphenylboron anion. More recently, benzyl sodium [26], along with metal enolate monofunctional initiators [27,28,29] such as sodium benzanilide and sodium deoxybenzoin, were developed to promote the anionic polymerization with characteristics of living reactions. More complex structures, such as star polymers, star-block and miktoarm star copolymers, were prepared by anionic polymerization high vacuum techniques [30].

The second approach was developed in 1991 by Novak’s Group. They developed a “living” coordination polymerization methodology based on titanium catalysts, leading to well-defined homopolymers [31] and block copolymers [32]. More specifically, organotitanium (IV) complexes of the type TiCl_3_(OCH_2_CF_3_) and CpTiCl_2_L (Cp = cyclopentadiene and L= -OCH_2_CF_3_, -N(CH_3_)_2_, -CH_3_) [33] were initially utilized. The replacement of one of the chlorine atoms with the bulkier and more electron-donating Cp group reduces the Lewis acidity of the titanium center and the polymerization of HIC proceeds in a slower but more controlled manner. Polymers having controlled molecular weights and narrow molecular weight distributions were obtained at high yields, with polymerizations conducted at room temperature. Following this methodology, several isocyanates were polymerized. Furthermore, diblock and triblock copolymers of poly(ethylene oxide) and poly(*n*-hexyl isocyanate), PEO-*b*-PHIC [34] and PHIC-*b*-PEO-*b*-PHIC [35], along with triblock copolymers having poly(dimethyl siloxane), PDMS, middle block and PHIC end blocks, PHIC-*b*-PDMS-*b*-PHIC [36], were synthesized. Using the same chemistry and in combination with anionic and atom transfer radical polymerization techniques, complex macromolecular architectures based on PHIC, such as block, graft, block-graft and miktoarm star copolymers and molecular brushes, were synthesized [37,38,39,40,41,42,43]. Several other applications of the organotitanium polymerization of isocyanates have been reported in the literature [44,45].

In this study, we report the synthesis of homopolymers, statistical and block copolymers based on n-hexyl isocyanate, HIC, and 3-(triethoxysilyl)propyl isocyanate, TESPI, by using a chiral, half-titanocene complex as initiator. The copolymers were characterized by nuclear magnetic resonance spectroscopy and size exclusion chromatography, and their thermal properties were investigated employing Thermogravimetric Analysis, TGA and Differential Thermogravimetry, DTG, measurements. The purpose of this work was to have a system that could be able to form cross-linked structures through the controlled hydrolysis of the alkoxysilyl groups [46,47]. In addition, these materials are able to react with certain surfaces or nanoparticles bearing hydroxyl groups in order to achieve the chemical modification of these surfaces or nanoparticles by grafting hydrophobic helical polymer chains onto them [48,49,50,51].

## 2. Materials and Methods

### 2.1. Materials

CpTiCl_3_ (Aldrich Europe, Buchs, Switzerland. 97%), hexanes, tetrahydrofuran and (S)-2-butanol (Aldrich, 99%) were used as received. n-Hexyl isocyanate, HIC, (Aldrich, 98%) and 3-(triethoxysilyl)propyl isocyanate TESPI, (Aldrich, 95%) were dried over CaH_2_ and 4,4′- methylene-bis(phenyl isocyanate) overnight and distilled under reduced pressure. Acetonitrile was dried over CaH_2_ and 4,4′-methylene-bis(phenyl isocyanate) overnight and distilled. Toluene was dried over CaH_2_ overnight and distilled. Ethanol and triethylamine were dried over Na and distilled. The initiator CpTiCl_2_(O-(S)-2-Bu) was synthesized according to previously reported protocols [52] and stored in the glove box. All synthetic procedures were performed under an inert atmosphere, employing standard Schlenk and glove box techniques [53]. The glassware used for the polymerization procedures was flame-dried under vacuum. Typical synthetic routes are presented below.

### 2.2. Synthesis of Poly[3-(Triethoxysilyl)propyl Isocyanate], PTESPI

In a 50 mL flask, [CpTiCl_2_(O-(S)-2-Bu)] (0.0370 g, 0.14 mmol) was dissolved in toluene (0.5 mL). 3-(Triethoxysilyl) propyl isocyanate, TESPI, (2.7 mL, 10.90 mmol) was added to the yellow solution. After 20 h, ethanol (1 mL) and toluene (1.5 mL) were added to the viscous solution. The solution became faint yellow immediately. The content of the Schlenk flask was transferred to a 50 mL Schlenk flask containing acetonitrile (20 mL). The white solid was separated from the yellow solution by filtration. The white polymer was dried under vacuum for 24 h. The polymer was soluble in toluene, tetrahydrofuran, chloroform and hexanes. Vigorous stirring for a few seconds is needed in order to be fully dissolved. The solution was colorless and clear.

Yield: 37%.

### 2.3. General Synthesis of Statistical Copolymers of HIC and TESPI, PHIC-Stat-PTESPI

In a 50 mL flask, [CpTiCl_2_(O-(S)-2-Bu)] was dissolved in toluene (1.0 mL). TESPI and HIC were added to the yellow solution. The copolymerization reaction was allowed to take place at room temperature. After 20 h, ethanol (1 mL) and toluene (5 mL) were added to the viscous solution. The solution became faint yellow immediately. The content of the Schlenk flask was transferred to a 100 mL Schlenk flask containing acetonitrile (25 mL). The white gel was separated from the yellow solution by removing the liquid phase. The faint yellow polymer was dried under vacuum for 24 h.

The amounts of each monomer and half-titanonocene complex for each sample are given in Table 1.

### 2.4. General Synthesis of Block Copolymers of HIC and TESPI, PTESPI-b-PHIC

As an example, the synthesis of the block copolymer B1 is described:

In a 50 mL flask, [CpTiCl_2_(O-(S)-2-Bu)] (0.0345 g, 0.13 mmol) was dissolved in toluene (0.5 mL). TESPI (0.5 mL, 2.01 mmol) was added to the yellow solution. After 1 h, HIC (1.0 mL, 6.86 mmol) was added. After 4 h, ethanol (0.5 mL) and toluene (2.0 mL) were added to the viscous solution. The solution became faint yellow immediately. The content of the flask was transferred to a 50 mL Schlenk flask containing acetonitrile (10 mL). The white crude solid product was partially soluble in toluene, tetrahydrofuran and chloroform due to the presence of insoluble inorganic compounds in the reaction mixture. Therefore, it was filtered through a Por.4 sintered glass funnel. The white polymer was dried under vacuum for 24 h. The polymer:

NMR Ratio (mol): 84% HIC and 16% TESPI.

Yield: 46%

Similar procedures were adopted for the remaining samples. More details are given in Table 2.

### 2.5. Characterization Techniques

The polymers were characterized by size exclusion chromatography, SEC, and ^1^H-NMR spectroscopy. SEC was performed on a modular instrument consisting of a Waters model 510 pump, a Waters model U6K sample injector, a Waters model 410 differential refractometer, a Waters model 486 UV spectrophotometer and a set of 5 μ-Styragel columns. The columns were housed in an oven thermostated at 40 °C. Tetrahydrofuran was the carrier solvent at a flow rate of 1 mL/min. The system was calibrated with eight polystyrene, PS, standards having molecular weights in the range of 1000 to 500,000.

The ^1^H NMR measurements were recorded on a Bruker Avance Neo (Billerica, MA, USA) V3–400 MHz spectrometer at room temperature in deuterated chloroform (CDCl_3_).

The thermal stability and the kinetics of thermal decomposition of the copolymers were studied by Thermogravimetric Analysis, TGA, employing a Q50 TGA model from TA Instruments (New Castle, DE, USA). The samples were placed in a platinum pan and heated up to 600 °C in a 60 mL/min flow of nitrogen at heating rates of 3, 5, 7, 10, 15 and 20 °C/min.

The optical properties of the copolymers were investigated via circular dichroism. CD spectra were recorded in hexane solutions using a Jasco J-815 CD spectrometer (Easton, MD, USA).

## 3. Results and Discussion

### 3.1. Polymerization of TESPI via Coordination Polymerization

The efficient polymerization of TESPI, employing CpTiCl_2_(O-(S)-2-Bu) as initiator, was confirmed using typical experimental conditions as in the case of the polymerization of HIC. The SEC trace of the polymer is given in Figure S1 of the Supporting Information Section, SIS. A typical symmetric peak of very low dispersity was obtained, meaning that the polymerization reaction is well controlled, as in the case of the polymerization of HIC. The number average molecular weight M_n_ value was equal to 15.9 × 10^3^, whereas the dispersity Ð was equal to 1.13. In order to achieve this result, the reaction yield was allowed to be less than 50%. Patten and Novak [31,33] reported the reversibility of the polymerization reaction of isocyanates with organotitanium (IV) catalysts. To avoid depolymerization and achieve high yields, the reaction should be conducted in bulk or at very high concentrations. Under these conditions, the viscosity of the polymerization solution becomes extremely high, and therefore, the dispersity of the polymer is drastically increased, and several side reactions may take place. To avoid all these drawbacks, the polymerization was allowed to take place up to moderate yields. For the present sample, the yield was 37%.

The synthesis of the desired product was also confirmed by ^1^H NMR spectroscopy, as shown in Figure S2 of the SIS. The characteristic peaks from the ethoxysilyl side groups are obvious in the spectrum, located at 1.19 ppm (-Si-OCH_2_-CH_3_, 9 protons) and 3.80 ppm (-Si-OCH_2_-CH_3_, 6 protons). All the signals are in agreement with the NMR data reported for the homopolymer, as synthesized by anionic polymerization techniques [23].

Circular dichroism, CD, measurements were recorded in the homopolymer to study the conformation of the polymer chain, as shown in Figure S3 of the SIS. In previous studies, polymerization of HIC with the same half-titanocene complex optically active helical polymers were obtained [52]. In the present case, the same conclusion was not confirmed. This is probably due to the steric hindrance introduced by the bulky side chain of TESPI. Thus, the polymer adopts a more random configuration in space. Finally, no change in conformation was observed upon changing the temperature up to 55 °C.

### 3.2. Statistical Copolymers

A set of five copolymers of HIC and TESPI were prepared in order to calculate the monomer reactivity ratios. Different feed ratios were involved in each copolymerization (monomer molar ratios HIC/TESPI: 80/20, 60/40, 50/50, 40/60 and 20/80). Different copolymers are denoted by the various feed molar ratios of the monomers, e.g., sample 20/80 indicates the copolymer was synthesized by using 20% HIC and 80% TESPI as molar feed composition. The copolymerization procedure was monitored by SEC and ^1^H-NMR spectroscopy.

**Figure 1:**
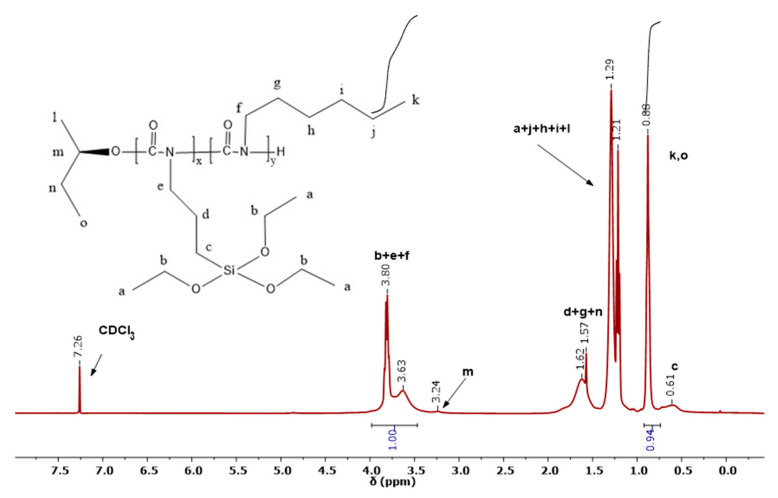
400 1H NMR spectrum of the statistical copolymer 80/20 in CDCl3.

The ^1^H-NMR spectrum of the statistical copolymer 80/20 is shown in Figure 1. The two peaks at 3.5–4.0 ppm are assigned to protons (b), (e) and (f). The single peak at 0.98 ppm corresponds to the three methyl protons (k) of HIC and the three (o) protons of the end-group coming from the initiator complex. In addition, the single peak at 0.61 ppm is due to (c) protons of TESPI. The multiple peaks around 1.2 ppm belong to the protons (a), (j), (h), (i) and (l), whereas the peak at 1.6 ppm is assigned to protons (d), (g) and (n). Finally, the small peak at 3.24 ppm is due to the (m) protons. From the integration of the peaks at 3.5–4.0 ppm and 0.9 ppm, the composition of the copolymer can be calculated.

**Figure 2:**
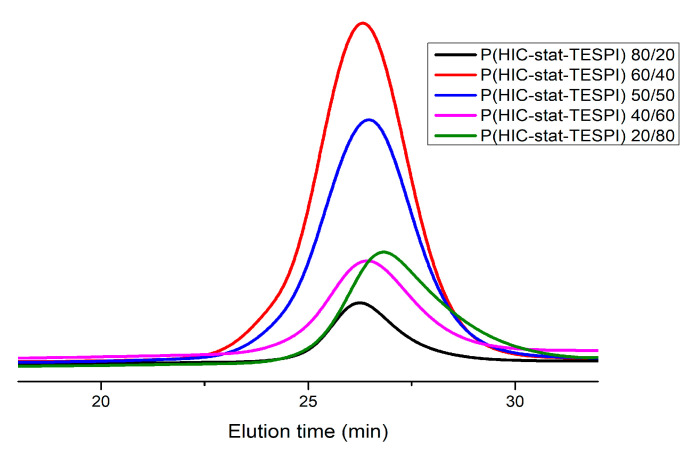
SEC traces of the statistical copolymers in THF.

The molecular characteristics of the statistical copolymers, derived from SEC measurements, along with their composition by NMR spectroscopy, are given in Table 3. The SEC traces of the statistical copolymers, shown in Figure 2, reveal the presence of symmetrical peaks having relatively low dispersity values. This was achieved since the conversions of the copolymerization reactions were relatively low, meaning that under these experimental conditions, the copolymerization procedure is well controlled. The low conversions were also desirable in order to obey the copolymerization equation and, thus, be able to apply the linear methods for the calculation of the monomer reactivity ratios [54], as will be described below.

Circular dichroism data from the statistical copolymers do not confirm the existence of helical conformation for the copolymeric chains. This is due to the random arrangement of the monomer units along the macromolecular chain and the steric hindrance introduced by the bulky side chain of TESPI, which facilitates a more planar conformation. Finally, no change in conformation was observed upon changing the solution temperature.

#### Reactivity Ratios

The monomer reactivity ratios were estimated using the well-documented Fineman–Ross [55], FR, inverted Fineman–Ross [55], inv-FR, Kelen–Tüdos [56] (KT) and extended Kelen–Tüdos [56], ext-KT, graphical methods, along with the computer program COPOINT [57].

The FR method, employed for the calculation of the reactivity ratios r_HIC_ and r_TESPI_, corresponding to the HIC and TESPI monomers, is based on the following equations:

$$G=Hr_{HIC}-r_{TESPI}$$

with $G=\frac{X(Y-1)}{Y}$, $H=\frac{X^{2}}{Y}$

and $X=\frac{{Μ}_{HIC}}{{Μ}_{TESPI}}$, $Y=\frac{d\left[M_{HIC}\right]}{d\left[M_{TESPI}\right]}$

where M_HIC_ and M_TESPI_ are the feed monomer composition, and dM_HIC_ and dM_TESPI_ are the final copolymer composition, as measured by the NMR spectra. The linear equation between G and H can be applied for the calculation of r_HIC_ and r_TESPI_ as the slope and the intercept of the plot, respectively.

The inv-FR method is obtained from the rearrangement of the data of the FR equation and is described by the following relationship:

$$\frac{G}{H}=r_{HIC}-\frac{1}{H}r_{TESPI}$$

Considering the linear plot of G/H vs. 1/H, the r_HIC_ can be obtained from the intercept, whereas the r_TESPI_ from the slope of the graph.

A different approach was proposed by Kelen and Tüdos in order to provide a better accuracy for the determination of the reactivity ratios. For this purpose, a new arbitrary constant (α) was introduced into the FR equation. The constant is equal to $\sqrt{H_{min}H_{max}}$, with H_min_ and H_max_ being the minimum and maximum values of H, respectively. The Kelen–Tüdos equation is given below:

$$\eta =\left(r_{HIC}+\frac{r_{TESPI}}{\alpha }\right)\xi -\frac{r_{TESPI}}{\alpha }$$

With $\eta =\frac{G}{\alpha +H}$ and $\xi =\frac{H}{H+\alpha }$

The plot of η vs. ξ is a straight line, yielding −r_TESPI_/α and r_HIC_ as intercepts on extrapolation tο ξ = 0 and ξ = 1, respectively.

The above-mentioned equations can only be employed for sufficiently low copolymerization conversions (ideally < 10%) in order to satisfy the copolymerization equation. However, the extended KT method can be applied for higher yields (up to 50%) since it takes into consideration the composition changes both in the reaction mixture of monomers and in the resulting copolymer. For this purpose, a new conversion-dependent parameter (z) is introduced, given by the equation:

$$z=\frac{log(1-\zeta_{HIC})}{log(1-\zeta_{TESPI})}$$

and the previous parameters G, H are redefined as:

$$G=\frac{Y-1}{a}\;and\;H=\frac{Y}{z^{2}}$$

The parameters ζ_TESPI_ and ζ_HIC_ are given by the following equations:

$$\zeta_{TESPI}=w\left(\frac{\mu +X}{\mu +Y}\right)$$

$$\zeta_{HIC}=\left(\frac{Y}{X}\right)\zeta_{TESPI}$$

where μ is the ratio of the molecular weight of PTESPI to the molecular weight of HIC, and w is the conversion of the copolymerization reactions.

Among these linear methodologies, the KT and ext-KT methods provide reactivity ratio values with relatively higher accuracy. However, they still are susceptible to statistical limitations, which are inherent more or less to all linear least-square approaches. To minimize these limitations, non-linear methodologies can be employed. Several non-linear approaches have been developed in the literature [54]. Among them, the COPOINT computer program is frequently employed. This software is based on non-linear least-squared difference procedures. COPOINT is a rather simple program that numerically integrates the differential copolymerization equations applied by the user and fits them to the experimental composition data. The copolymerization parameters are determined after minimizing the sum of the square difference between the measured and calculated polymer compositions. COPOINT also evaluates the statistical error of the sum and provides the user with a probable error range for the estimated parameters.

**Figure 3:**
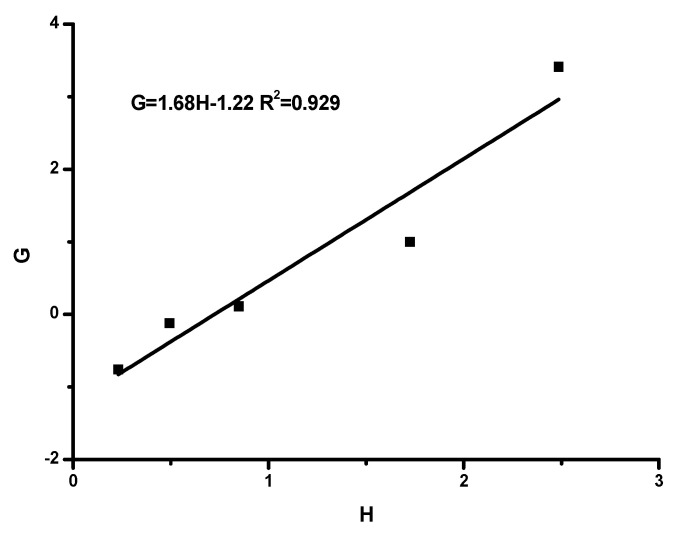
FR plot.

**Figure 4:**
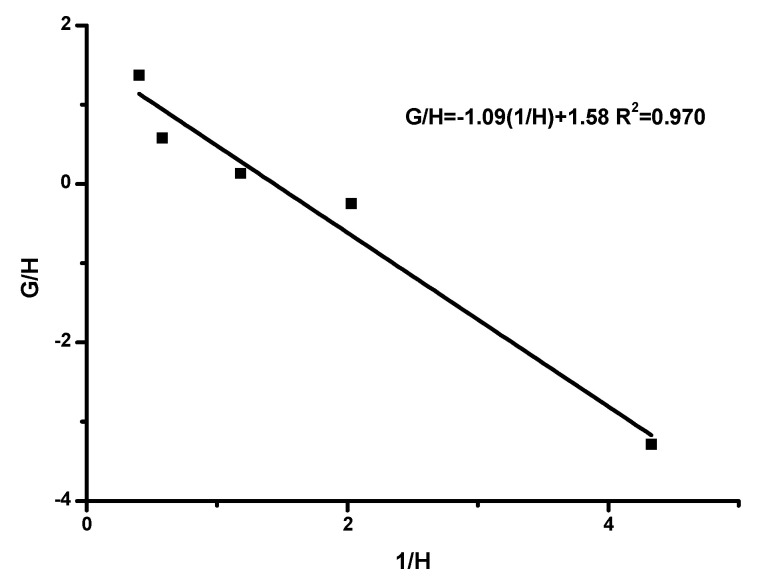
inv-FR plot.

**Figure 5:**
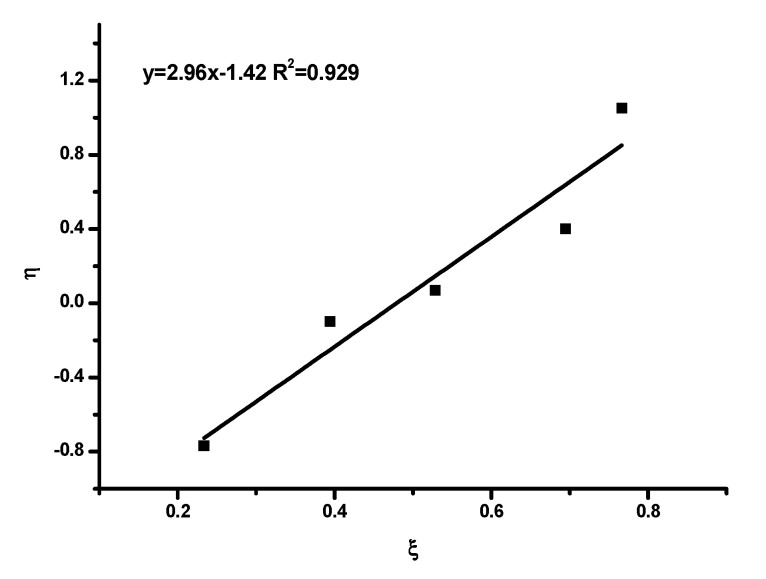
KT plot.

**Figure 6:**
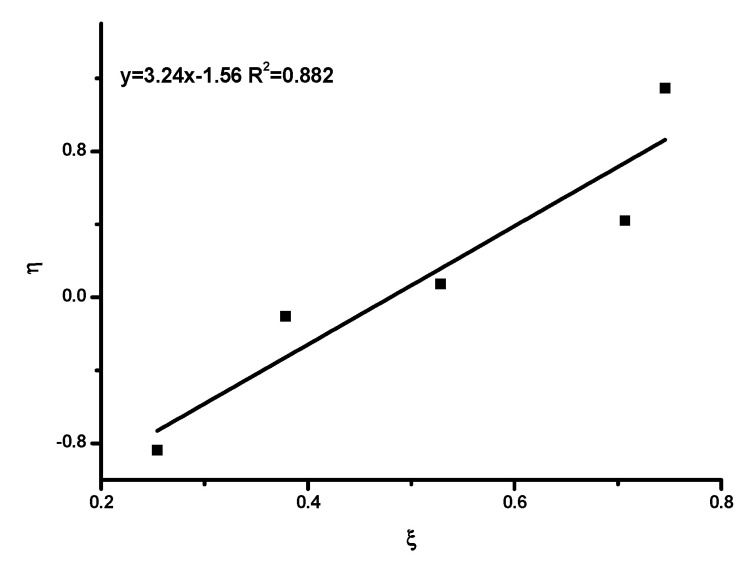
ext-KT plot.

The copolymerization data are given in Table S1 of the SIS, and the associated graphs are given in Figure 3, Figure 4, Figure 5 and Figure 6. The analysis was based on the terminal model [54,58]. The similarity of the chemical nature of the two isocyanate monomers, along with the very good linearity of the plots, support the conclusion that the terminal model better suites this copolymerization reaction and further suggests that the copolymerization follows the conventional copolymerization kinetics. According to the terminal model, the propagation reaction is governed only by the nature of the monomer and of the terminal unit of the growing polymer chain. The monomer reactivity ratios, r_HIC_ and r_TESPI_, calculated by all the aforementioned methods, are reported in Table 4.

The different calculation methods yielded similar results for the reactivity ratios. The values of both HIC and TESPI are greater than unity or very close to that, while the reactivity ratio of HIC is considerably higher than that of TESPI. These findings reveal that both monomers tend to homopolymerize, finally forming multiblock copolymers. Since the reactivity ratio of HIC is higher than that of TESPI, the monomer sequences of the HIC monomer units are longer than those of TESPI. To confirm these conclusions, the statistical distribution of the dyad monomer sequences M_HIC_-M_HIC_, M_HIC_-M_TESPI_, and M_TESPI_-M_TESPI_ was calculated according to the Igarashi equations [59]:

Χ=φHIC−2φHIC1−φHIC1+2φHIC−12+4rHICrTESPIφHIC1−φHIC12

Υ=1−φHIC−2φHIC1−φHIC1+2φHIC−12+4rHICrTESPIφHIC1−φHIC12

Ζ=4φHIC1−φHIC1+2φHIC−12+4rHICrTESPIφHIC1−φHIC12

where X, Y and Z are the mole fractions of the M_HIC_-M_HIC_, M_TESPI_-M_TESPI_ and M_HIC_-M_TESPI_ dyads in the copolymer, respectively, whereas φ_HIC_ is the HIC mole fraction in the copolymer. Τhe mean sequence lengths, μ_HIC_ and μ_TESPI_, were also calculated using the following equations [54]:

$$\mu_{HIC}=1+r_{HIC}\frac{\left[M_{HIC}\right]}{\left[M_{TESPI}\right]}$$

$$\mu_{TESPI}=1+r_{TESPI}\frac{\left[M_{TESPI}\right]}{\left[M_{HIC}\right]}$$

**Figure 7:**
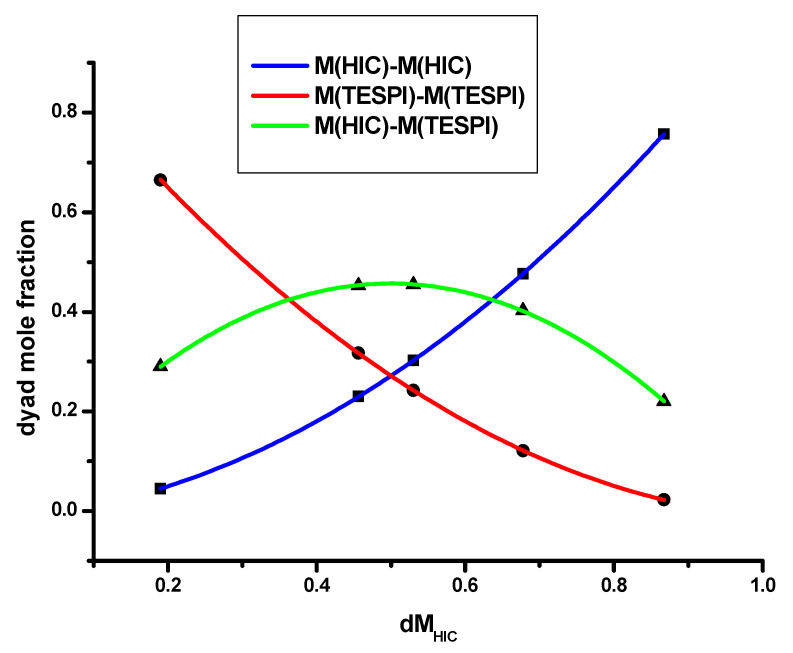
Dyad mole fraction versus the HIC mole fraction of the statistical copolymers.

The results are provided in Table 5, and the plot of the dyad mole fraction versus the HIC mole fraction is given in Figure 7. Τhe results confirm the conclusion drawn by the reactivity ratios.

### 3.3. Block Copolymers

The synthesis of block copolymers PHIC-*b*-PTESPI was conducted by sequential addition of monomers, which always starts from the polymerization of TESPI. TESPI is a bulkier monomer than HIC with a lower rate of polymerization, as was confirmed by the calculation of the reactivity ratios. Therefore, the polymerization of TESPI was conducted first for the following reasons: (a) The polymerization of TESPI as the second monomer would be very difficult due to the low rate of polymerization of this monomer and the fact that the reaction had to be promoted in a viscous medium after the polymerization of the HIC as the first monomer. These conditions would lead to a loss of control of the molecular characteristics of the block copolymers and minimal incorporation of TESPI monomer units into the final product. (b) It is well reported that the polymerization yield is not allowed to reach very high levels in order to control the molecular characteristics of the produced polyisocyanate. If HIC was the first monomer for polymerization, the subsequent addition of TESPI would result in a second block, which would actually be a statistical copolymer containing not only TESPI but HIC monomer units as well coming from the unreacted quantity of the HIC monomer after the polymerization of the first block. Therefore, the final product would have increased chemical heterogeneity. When TESPI is polymerized first, the remaining quantity of the monomer will be very difficult to further react after the addition of HIC due to its low polymerization rate compared to that of HIC. Consequently, the purity of the final product is highly improved.

**Figure 8:**
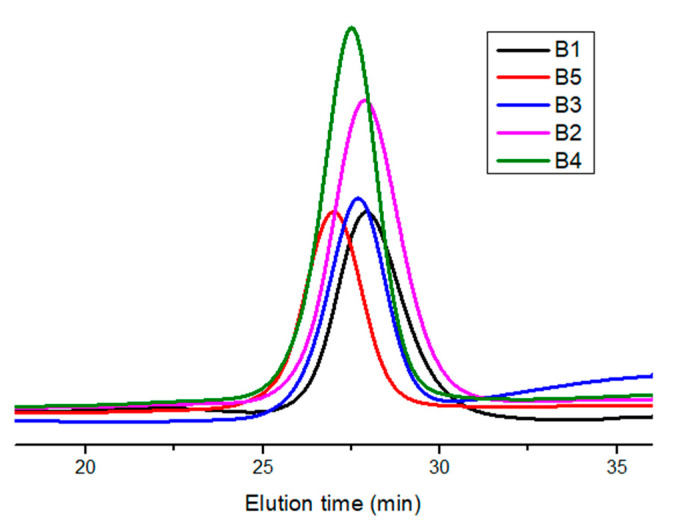
SEC traces of the block copolymers in THF.

The samples are denoted by the letter B followed by a number differentiating the various samples. The synthetic procedure was monitored by SEC. The characteristic traces of all samples are given in Figure 8, whereas the molecular characteristics of the block copolymers are in Table 6. In all cases, the peaks were symmetrical, and the samples had very low dispersity values, indicating that the copolymerization reaction was very well controlled for all the samples. The conversions were relatively high but not quantitative (always lower than 80% for both monomers). Relatively low molecular weight samples were targeted in order to have lower viscosity solutions during the copolymerization reaction, thus avoiding termination reactions and achieving better control over the molecular characteristics. It was not easy to take samples for SEC analysis of the first block since sampling from a very viscous media would result in termination reactions.

**Figure 9:**
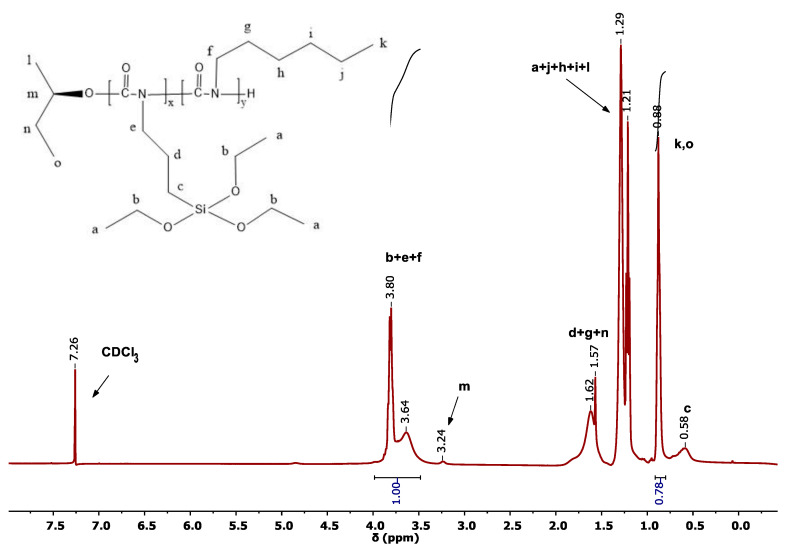
400 MHz ^1^H NMR spectrum of the sample B1 in CDCl_3_.

The copolymer compositions were calculated by NMR spectroscopy, as analyzed previously in the case of the statistical copolymers. A characteristic spectrum is given in Figure 9. The compositions of the samples were very close to the stoichiometry employed for the copolymer synthesis, confirming the high control, which was promoted during the copolymerization reaction.

**Figure 10:**
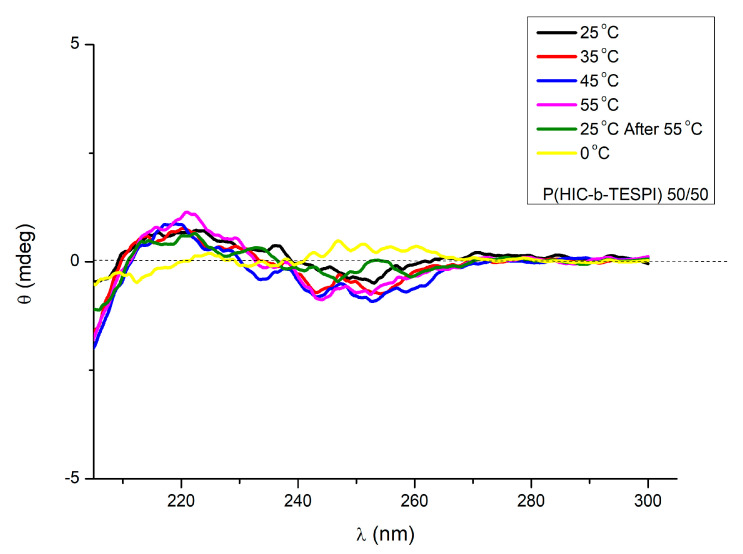
CD spectra of the sample B2 at various temperatures.

The chirality of the block copolymer chains was verified by their CD spectra, as shown in Figure 10. The chirality originates from the PHIC blocks since PTESPI adopts a more flexible and random conformation, as was indicated by the absence of signals in the PTESPI spectrum. The copolymer exhibits a Cotton effect at 255 nm due to the n-p* transitions of the amide chromophore, which is negative. This leads to the conclusion that the helix is left-handed (M). At shorter wavelengths (205 nm), an exciton couplet was observed due to the arrangement of the chiral amide linkages along the main chain. The stability of the helical structure in solution upon increasing the temperature was also studied [60]. The structure changes progressively, from rigid rod to coil, with the temperature increase, in agreement with the literature. This behavior is reversible in heating and cooling cycles. This phenomenon is not readily observed in a low molecular weight polymer, meaning that the helical structure remains intact at least up to 55 °C, as proven in previous studies [52].

### 3.4. Thermal Decomposition Studies

#### 3.4.1. Homopolymers

**Figure 11:**
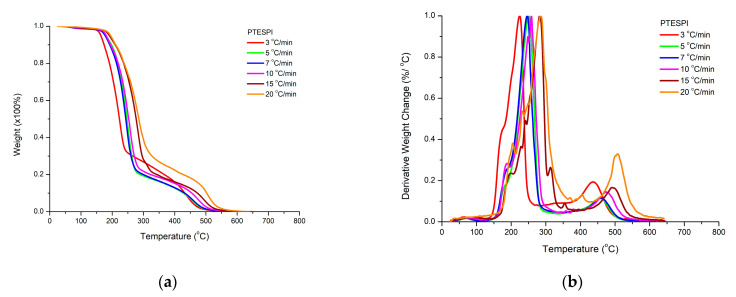
TGA (**a**) and DTG (**b**) plots for PTESPI at various heating rates.

**Figure 12:**
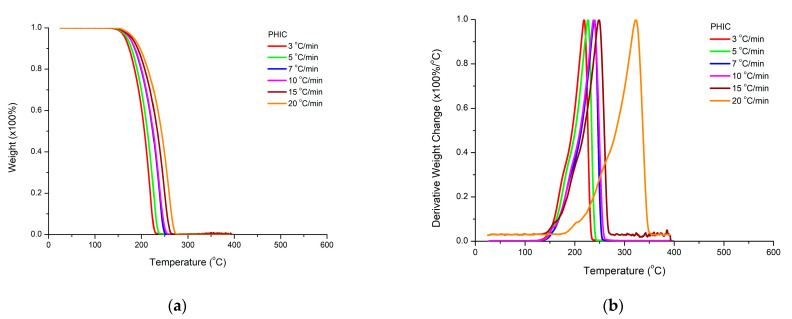
TGA (**a**) and DTG (**b**) plots for PHIC at various heating rates.

The thermal stability of the two homopolymers, PHIC and PTESPI, was studied by TGA and DTG measurements under different heating rates. The results are presented in Figure 11 and Figure 12.

The DTG plots for PHIC reveal the presence of a single decomposition peak at all heating rates. However, the presence of a small shoulder at lower temperatures is obvious. Despite this observation, the mechanism of thermal decomposition of PHIC is not very complex and, in addition, the homopolymer is considered to be thermally unstable since the decomposition process is initiated at 150 °C. A slightly different behavior was confirmed for PTESPI. A major decomposition step is present as well, accompanied by a small shoulder at lower temperatures, as in the case of PHIC. However, a second degradation peak is also observed at much higher temperatures. This event may be attributed to the thermal degradation of inorganic silicon-based residues produced from the decomposition of the side groups of the PTESPI monomer units. In addition, PTESPI is considered to be a more thermally stable polymer compared to PHIC since both the initiation and the completion of the degradation process are located at higher temperatures (from 10 to 20 °C) than PHIC chains.

From these results, it is reasonable to conclude that the nature of the side group of polyisocyanates may affect the thermal stability of the polymers despite the fact of the chemical similarity of the main polymeric chain for all polyisocyanates. Coordination polymerization techniques have been performed by our group for the homopolymerization of 2-chloroethyl isocyanate, ClEIC [61], and 2-phenylethyl isocyanate, PEIC [62], as well. The thermal stability of the respective homopolymers follows the order: PClEIC < PHIC < PTESPI < PPEIC. The thermally stable aromatic ring in PPEIC offers higher thermal stability to the respective homopolymer compared to the other polyisocyanates. PClEIC was found to be the more thermally sensitive polymer. The thermal elimination of the side chlorine groups may be associated with the formation of reactive radicals, which evidently promotes the further decomposition of the polymeric chains. More details will be provided in future publications.

The rate of heating affects the thermal decomposition by increasing the temperatures of decomposition for the same sample. This result confirms that the thermal degradation process is also a kinetic phenomenon. The higher the heating rate, the slower the response of the material to the induced change.

#### 3.4.2. Statistical Copolymers

**Figure 13:**
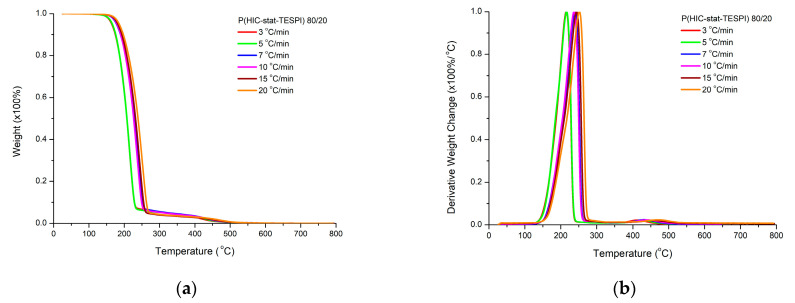
TGA (**a**) and DTG (**b**) plots for the statistical copolymer 80/20 at various heating rates.

The thermal degradation of the statistical copolymers 80/20, 60/40 and 50/50 was studied, and the results are displayed in Figure 13 and Figures S4 and S5 of the SIS.

The thermal decomposition profiles of the statistical copolymers combine the characteristics of both the PHIC and PTESPI homopolymers degradation behavior. A single major decomposition step prevails, having a small shoulder at lower temperatures, as in the case of the PHIC and PTESPI homopolymers. A second small peak is also visible, coming from the presence of the sequences of the TESPI monomer units. This peak is not so pronounced as in the case of the PTESPI homopolymer. However, the peak becomes more intensive upon increasing the TESPI content of the statistical copolymer.

The temperature at the maximum rate of thermal degradation is decreased upon increasing the content in HIC monomer units. This result is reasonable, taking into account the increased thermal stability of the PTESPI homopolymer compared to that of PHIC. In addition, this temperature value is closer to that found for the PTESPI sample, even in the case where the content in HIC monomer units is higher in the copolymer. Finally, the range of decomposition temperatures is similar to that found for the PTESPI homopolymer. These results indicate that the presence of TESPI units offer greater influence than the HIC units to the statistical copolymers, improving their thermal stability.

#### 3.4.3. Block Copolymers

**Figure 14:**
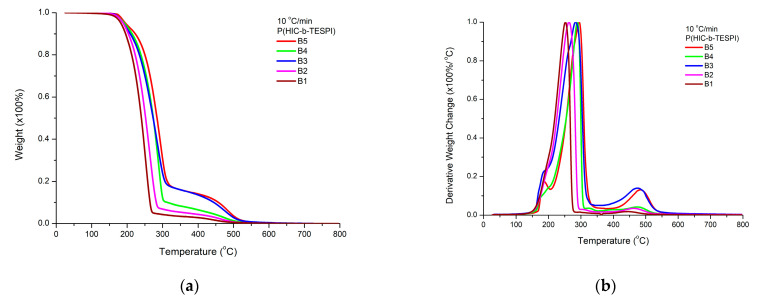
TGA (**a**) and DTG (**b**) plots for the block copolymers at the heating rate of 10 °C/min (samples B5, B4, B3, B2, B1 from top to bottom).

The thermal stability of the block copolymers was examined at a heating rate of 10 °C/min. The TGA and DTG plots are given in Figure 14.

The degradation profiles of the blocks are somewhat different than those of the statistical copolymers, thus manifesting the effect of the macromolecular architecture on the thermal stability. The main decomposition peak is similar to that observed for the statistical copolymers as well. However, the peak at higher temperatures (450–500 °C), attributed to the PTESPI block, is clearly observed, and its contribution becomes progressively more pronounced upon increasing the PTESPI content of the block copolymers. In addition, the shoulder at lower temperatures, which is especially obvious in the case of the PHIC homopolymer, is clearly present in the case of the block copolymers at the range of 150–200 °C. This shoulder becomes a distinct peak for the sample having the highest PTESPI content. In this case, the temperature of the main decomposition peak is increased due to the increased thermal stability of PTESPI compared to PHIC. Therefore, the shoulder at lower temperatures is further separated from the main degradation event and becomes obvious as a separate small peak. These results clearly indicate that the two blocks have an independent decomposition profile without being affected by each other to a great extent.

The temperature at the major degradation peak is closer to the temperature corresponding to the PTESPI homopolymer than that of PHIC, as in the case of the statistical copolymers. This conclusion indicates that the presence of PTESI offers increased thermal stability to the block copolymers.

### 3.5. Kinetics of the Thermal Decomposition of the Homopolymers and the Statistical Copolymers

Several methodologies have been applied for the calculation of the activation energies, E_a_, of the thermal decomposition of polymeric substances [63] Among them, the well-established isoconversional Ozawa–Flynn–Wall (OFW) [64,65,66] and Kissinger–Akahira–Sunose (KAS) [67] methods play a dominant role. They can be applied using data of the TGA measurements without knowledge of the exact mechanism of degradation. In addition, they provide data for the E_a_ at each step of the decomposition process. They are based on the following equations:

$$(\mathrm{OFW}):\;ln\beta =ln\left[\frac{0.0048AEa}{g\left(a\right)R}\right]-1.0516\frac{Ea}{RT}$$

$$(\mathrm{KAS}):\;ln\frac{\beta }{T^{2}}=ln\left[\frac{AR}{g\left(a\right)Ea}\right]-\frac{Ea}{RT}$$

where α is the conversion, f(α) is the conversion function, g(α) is the integral conversion, β is the heating rate, T is the absolute temperature, R is the gas constant (R = 8.314 $\frac{J}{mol*K}$), A is the pre-exponential factor ($\frac{1}{min}$), and E_a_ is the activation energy ($\frac{kJ}{mol}$). Displaying lnβ versus $\frac{1}{T}$ or ln($\frac{\beta }{T^{2}}$) versus $\frac{1}{T}$, respectively, should emerge in lines with a slope that is directly proportional to the activation energy. Moreover, a single-step degradation reaction can be inferred if the determined activation energy values do not significantly change with different values of α.

**Figure 15:**
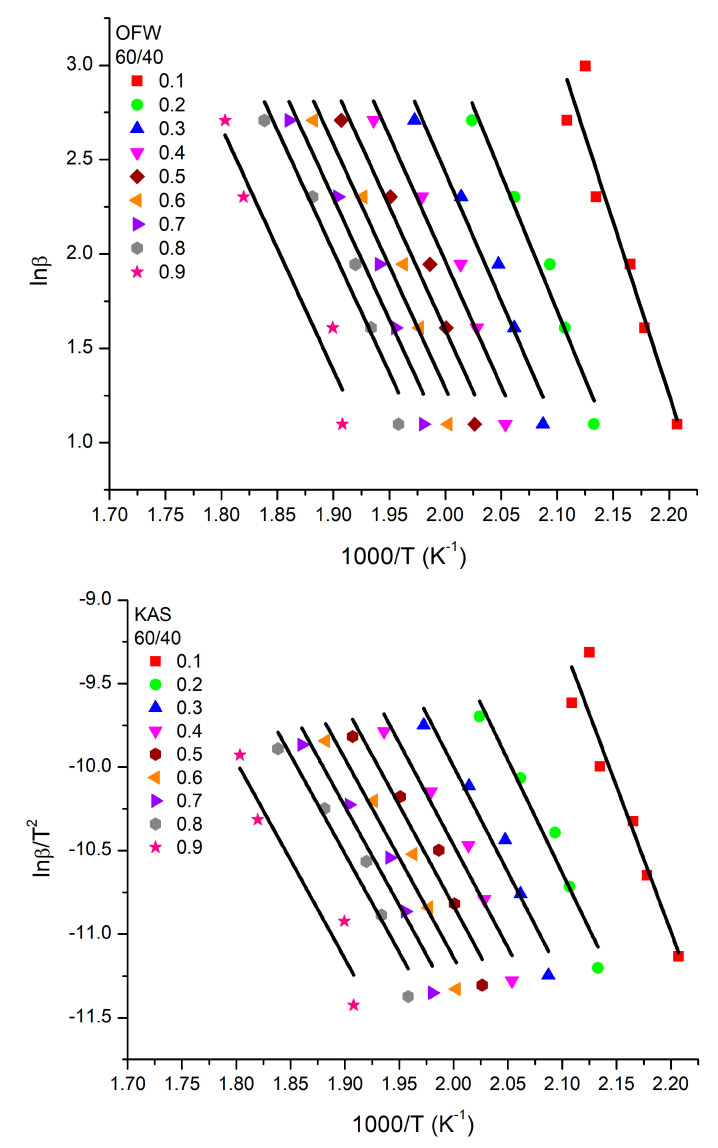
OFW and KAS plots for the statistical copolymer 60/40.

The OFW and KAS plots for the PHIC and PTESPI homopolymers, along with the 80/20, 60/40 and 50/50 statistical copolymers, are given in Figure 15 and Figures S6–S9 of the SIS, whereas the E_a_ values for each sample and for various conversions are provided in Table 7 and Table 8.

The results derived from the OFW and KAS methods for the homopolymers and the copolymers are similar, indicating that both approaches provide reliable data for the E_a_ values. These values are generally small for all samples and conversions, which means that a large energy barrier is not required for thermal degradation and that this family of polymers does not belong to the class of thermally stable polymers.

In particular, the E_a_ values of PTESPI are relatively smaller than those of PHIC. The differences are not huge since both homopolymers belong to the same family of polymers with a common main chain structure. These variations have to do with the presence of the side groups attached to the main chain [68,69]. The existence of these differences demonstrates that the thermal degradation mechanistically involves not only the cleavage of the main chain to smaller ones but also the cleavage of the side groups. These two events occur simultaneously, which is why the thermal degradation peaks from the DTG plots are not perfectly symmetrical and show shoulders in many cases. However, the E_a_ values do not appreciably change with the conversion, indicating that the degradation mechanism is relatively simple and mainly remains approximately the same throughout the thermal degradation.

In the case of the copolymers, the E_a_ values are generally between the values of the respective homopolymers but clearly much closer to those of the PHIC homopolymer. This is reasonable since the examined copolymers had a higher content in HIC monomer units compared to TESPI monomer units.

Among the various copolymers, the largest E_a_ values were observed for the sample 60/40. Probably for this particular sample, the maximum possible stabilization of the tertiary structure is achieved, meaning that a higher energy barrier needs to be overcome for the thermal degradation of the sample. Similar behavior, in the sense that some of the copolymers with an intermediate composition presented the maximum E_a_ value, has also been observed in the statistical copolymers of PHIC with poly(2-chloroethyl isocyanate), P(HIC-*stat*-ClEtIC) [61] and poly(2-phenylethyl isocyanate), P(HIC-*stat*-PEIC) [62].

It is known that the function g(α) depends on the conversion mechanism and its mathematical model [70]. Algebraic expressions of functions of the most common reaction mechanisms operating in solid-state reactions are presented in the SIS (Table S2). Rearranging the KAS equation, the following equation is obtained:

(1) $$ln\frac{g(a)}{T^{2}}=ln\left[\frac{AR}{\beta E_{a}}\right]-\frac{E_{a}}{RT}$$

**Figure 16:**
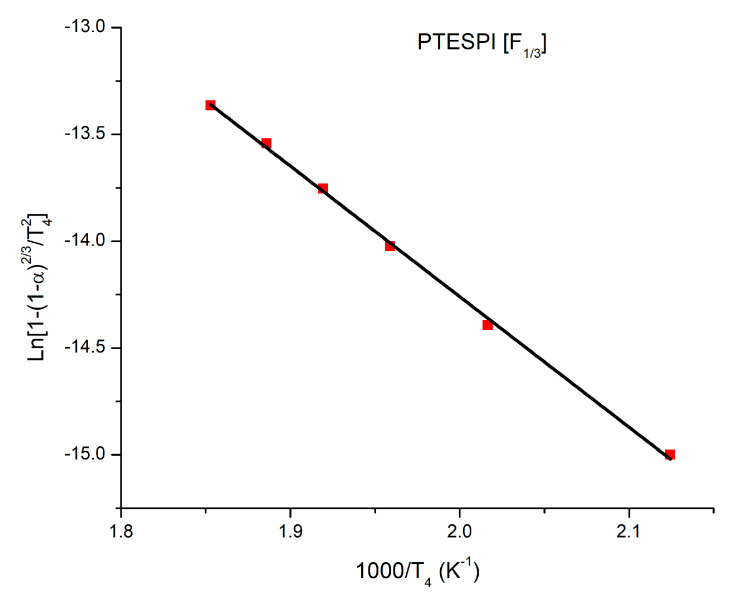
Plot of the chemical reaction [F_1/3_] model for PTESPI.

According to this equation, the ln[g(a)/T^2^] vs. 1000/T graphs are created for a certain value of β (for example, β = 10 °C/min) and for the various proposed models. The slopes of these plots are able to determine the E_a_ values, while the intercepts may lead to the calculation of the pre-exponential factors, A. The plot with the best linear fitting and the best agreement between the theoretical and the experimental E_a_ values, as determined by the OFW and KAS methods, represents the mathematical model or mechanism by which the thermal decomposition occurs. The plot with the best linearity and the best proximity of the E_a_ values for the homopolymer PTESPI is given in Figure 16. Taking into account this result, the PTESPI homopolymer degrades with the mechanism [F_1/3_] since the E_a_ values predicted by the model and experimentally obtained by the OFW and KAS approaches are identical. This mechanism belongs to the category of chemical reactions. The corresponding diagram is of the theoretical model with E_a_ equal to 50.80 J/mol and a pre-exponential factor equal to 8.00 min^−1^ (Figure 16).

## 4. Conclusions

Coordination polymerization employing an optically active half-titanocene complex was efficiently applied for the synthesis of poly[3-(triethoxysilyl)propyl isocyanate], PTESPI and the subsequent preparation of well-defined statistical and block copolymers with poly(n-hexyl isocyanate). All samples were characterized by nuclear magnetic resonance, NMR, spectroscopy and size exclusion chromatography, SEC. The terminal model was applied to calculate the monomer reactivity ratios of the statistical copolymers, employing both linear graphical methods and the computer program COPOINT. It was found that the formation of multiblock copolymers is favored, having longer sequences of HIC monomer units. The dyad monomer sequences and the mean sequence lengths were also calculated, confirming the previous conclusions. Well-defined block copolymers PTESPI-b-PHIC with controlled molecular weights and low dispersities were synthesized by sequential addition of monomers, starting from the polymerization of TESPI. It was found by the CD spectra that PTESPI adopts a more flexible and random conformation in solution. However, in the case of the block copolymers, a negative Cotton effect at 255 nm was observed due to the helical structure, which is adopted by the PHIC block. The thermal stability and the kinetics of the thermal decomposition of the homopolymers, the statistical and the block copolymers were studied by Thermogravimetric Analysis, TGA, and Differential Thermogravimetry, DTG. The activation energy of this process was calculated, employing the isoconversional Ozawa–Flynn–Wall (OFW) and Kissinger–Akahira–Sunose (KAS) approaches. It was found that PTESPI is relatively thermally more stable than PHIC, in terms that the thermal degradation of PTESPI is completed at much higher temperatures than PHIC. In other words, the nature of the polymers’ side groups considerably affects the thermal stability of the polyisocyanate chains. The thermal decomposition of the statistical and block copolymers resembles that of the respective homopolymers.

## Figures and Tables

**Table 1:** Synthesis of the statistical copolymers PHIC-*stat*-PTESPI.

Ratio	80/20	60/40	50/50	40/60	20/80
[CpTiCl_2_(O-(S)-2-Bu)]	0.0528 g, (0.20 mmol)	0.0564 g, (0.21 mmol)	0.0568 g, (0.22 mmol)	0.0544 g, (0.21 mmol)	0.0529 g, (0.21 mmol)
TESPI	1.6 mL, (6.46 mmol)	2.8 mL, (11.31 mmol)	3.3 mL, (13.32 mmol)	3.7 mL, (14.94 mmol)	4.4 mL, (17.77 mmol)
HIC	3.8 mL, (26.08 mmol)	2.5 mL, (17.16 mmol)	1.9 mL, (13.04 mmol)	1.4 mL, (9,61 mmol)	0.6 mL, (4.11 mmol)
Yield	38%	39%	47%	41%	44%

**Table 2:** Synthesis of the block copolymers PTESPI-*b*-PHIC.

Sample	B1	B2	B3	B4	B5
[CpTiCl_2_(O-(S)-2-Bu)]	0.0345 g (0.13 mmol)	0.0293 g (0.11 mmol)	0.0349 g (0.14 mmol)	0.0273 g (0.11 mmol)	0.0285 g (0.12 mmol)
TESPI	0.5 mL (2.01 mmol)	0.8 mL (3.23 mmol)	1.0 mL (4.03 mmol)	1.1 mL (4.44 mmol)	1.30 mL (5.25 mmol)
HIC	1.0 mL (6.86 mmol)	0.7 mL (4.80 mmol)	0.5 mL (3.43 mmol)	0.4 mL (2.74 mmol)	0.2 mL (1.37 mmol)

**Table 3:** Molecular characteristics of the statistical copolymers.

Molar Monomer Ratio in Feed (HIC/TESPI)	^a^ M_w_ × 10^−3^	^a^ Ð	^b^ Mol Composition (HIC/TESPI)	^c^ Yield
80/20	22.1	1.17	87/13	38%
60/40	25.0	1.25	68/32	39%
50/50	23.6	1.26	53/47	47%
40/60	22.0	1.28	45/54	41%
20/80	11.8	1.36	19/81	44%

^a^ by SEC in CHCl_3_; ^b^ by ^1^H NMR in CDCl_3_; ^c^ Based on both monomers, after precipitation and purification, calculated gravimetrically.

**Table 4:** Reactivity ratios of HIC and TESPI in the statistical copolymers P(HIC-stat-TESPI).

Method	r_HIC_	r_TESPI_
F-R	1.68	1.22
IF-R	1.58	1.09
K-T	1.54	1.07
ext K-T	1.69	1.05
COPOINT	1.49 ^a^	0.95 ^b^

^a^: ±3.6% ^b^: ±1.99%, estimated error ranges from COPOINT measurements.

**Table 5:** Dyad sequences and mean sequence lengths of the statistical copolymers.

SAMPLE	M(HIC)–M(HIC)	M(TESPI)–M(TESPI)	M(HIC)–M(TESPI)	μ(HIC)	μ(TESPI)
20/80	0.04496	0.66496	0.29009	1.35	5.09
40/60	0.22981	0.31721	0.45299	1.96	2.48
50/50	0.30276	0.24256	0.45467	2.46	1.97
60/40	0.47609	0.12109	0.40283	3.84	1.50
80/20	0.75697	0.02277	0.22026	7.02	1.24

**Table 6:** Molecular characteristics of the block copolymers.

Sample	^a^ M_w_ × 10^−3^	^a^ Ð	^b^ Mole% Composition (HIC/TESPI)
B1	11.1	1.19	84/16
B2	12.2	1.19	65/35
B3	13.5	1.12	28/72
B4	14.7	1.14	26/74
B5	17.3	1.16	14/86

^a^ by SEC in CHCl_3_; ^b^ by ^1^H NMR in CDCl_3._

**Table 7:** Activation Energy (Ε_a_) values for the homopolymers and the statistical copolymers (KAS methodology).

Conversion	Ε_a_ (kJ/mol) PHIC	Ε_a_ (kJ/mol) PTESPI	Ε_a_ (kJ/mol) 50/50	Ε_a_ (kJ/mol) 60/40	Ε_a_ (kJ/mol) 80/20
0.1	133.78	73.58	68.11	144.34	117.54
0.2	116.64	67.09	98.78	123.32	106.75
0.3	107.00	62.55	54.31	109.91	100.04
0.4	95.58	60.48	88.30	104.05	96.75
0.5	100.54	58.65	86.52	101.26	94.86
0.6	97.39	48.19	85.52	99.82	93.74
0.7	96.06	61.86	85.28	99.30	85.23
0.8	94.71	57.05	85.35	99.02	94.16
0.9	93.42	27.77	86.19	78.53	101.64

**Table 8:** Activation Energy (Ε_a_) values for the homopolymers and the statistical copolymers (OFW methodology).

Conversion	Ε_a_ (kJ/mol) PHIC	Ε_a_ (kJ/mol) PTESPI	Ε_a_ (kJ/mol) 50/50	Ε_a_ (kJ/mol) 60/40	Ε_a_ (kJ/mol) 80/20
0.1	134.46	81.39	75.25	152.06	125.16
0.2	118.38	75.29	106.76	131.56	114.58
0.3	109.39	71.04	62.69	118.10	108.03
0.4	98.64	69.19	96.81	112.38	104.87
0.5	103.47	67.55	95.19	109.72	103.08
0.6	100.57	57.52	94.22	108.38	102.05
0.7	99.38	62.17	94.43	107.96	93.59
0.8	98.15	69.27	95.25	107.78	102.64
0.9	96.99	42.13	75.25	87.49	110.24

## Data Availability

Data will be made available on request.

## Supplementary Materials

The following supporting information can be downloaded at: https://www.mdpi.com/article/10.3390/polym15204113/s1. The following figures and tables are included in the supporting information section: Figure S1: SEC trace of PTESPI in THF; Figure S2: 400 MHz ^1^H NMR spectrum of PTESPI in CDCl_3_; Figure S3: CD spectrum of PTESPI in hexane for various temperatures; Figure S4: TGA and DTG plots for the statistical copolymer 60/40 at various heating rates; Figure S5: TGA and DTG plots for the statistical copolymer 50/50 at various heating rates; Figure S6: (OFW) and (KAS) plots for the statistical copolymer 80/20; Figure S7: (OFW) and (KAS) plots for the statistical copolymer 50/50; Figure S8: (OFW) and (KAS) plots for PTESPI; Figure S9: (OFW) and (KAS) plots for PHIC; Table S1: Copolymerization data for the statistical copolymers; Table S2: Models of thermal decomposition.
