# Supplementary material for: Synthesis and Characterization of Statistical and Block Copolymers of n-Hexyl Isocyanate and 3-(Triethoxysilyl) Propyl Isocyanate via Coordination Polymerization
Source: Polymers (Basel). 2023 Oct 17;15(20):4113. doi: 10.3390/polym15204113 (PMC10610266; doi:10.3390/polym15204113)
Supplement: Supplementary file 1 [file polymers-15-04113-s001.zip › polymers-2648919-supplementary.pdf]

Synthesis and Characterization of Statistical and Block Copolymers of n-hexyl Isocyanate and 3-(triethoxysilyl) Propyl Isocyanate via Coordination Polymerization

Maria Panteli, Dimitra Mantzara, Aikaterini Katara, Ioannis Choinopoulos * and Marinos Pitsikalis *

Industrial Chemistry Laboratory, Department of Chemistry, National and Kapodistrian University of Athens, Panepistimiopolis Zografou, 15771 Athens, Greece; mariap9655@gmail.com (M.P.); di.mitroula1997@hotmail.com (D.M.); katerinakatara01@gmail.com (A.K.)
* Correspondence: ichoinop@chem.uoa.gr (I.C.); pitsikalis@chem.uoa.gr (M.P.); Tel.: +30-210-727-4440 (M.P.)

Supporting Information Section

Table S1: Copolymerization data for the statistical copolymers,

A:HIC and B:TESPI

SAMPLE	M_A	dM_A	M_B	dM_B	X	Y	Gm	Hm	Gm/Hm	1/Hm	η	ξ
**20/80**	0.1888	0.1900	0.8112	0.8100	0.2327	0.2346	-0.7595	0.2309	-3.2888	4.3303	-0.7682	0.2336
**40/60**	0.3914	0.4563	0.6086	0.5437	0.6431	0.8392	-0.1232	0.4928	-0.2500	2.0291	-0.0985	0.3941
**50/50**	0.4946	0.5301	0.5054	0.4699	0.9786	1.1281	0.1111	0.8490	0.1309	1.1779	0.0692	0.5284
**60/40**	0.6556	0.6775	0.3444	0.3225	1.9036	2.1008	0.9975	1.7249	0.5783	0.5797	0.4018	0.6948
**80/20**	0.8011	0.8671	0.1989	0.1329	4.0277	6.5245	3.4103	2.4863	1.3716	0.4022	1.0513	0.7664

Table S2. Models of thermal decomposition

**No.**	**Model**	***g*(*α*)**	***f*(*α*)**	**Rate-determining mechanism**
1. Chemical process or mechanism non-involving equations				
1	$F_{1/3}$	$1-(1-\alpha)^{2/3}$	$(3/2)(1-\alpha)^{1/3}$	Chemical reaction
2	$F_{3/4}$	$1-(1-\alpha)^{1/4}$	$4(1-\alpha)^{3/4}$	Chemical reaction
3	$F_{3/2}$	$(1-\alpha)^{-1/2}-1$	$2(1-\alpha)^{3/2}$	Chemical reaction
4	F_{2}	$(1-\alpha)^{-1}-1$	$(1-\alpha)^{2}$	Chemical reaction
5	F_{3}	$(1-\alpha)^{-2}-1$	$(1/2)(1-\alpha)^{3}$	Chemical reaction
6	F_{4}	$(1-\alpha)^{-3}-1$	$(1/3)(1-\alpha)^{4}$	Chemical reaction
7	G_{1}	$1-(1-\alpha)^{2}$	$1/[2(1-\alpha)]$	Chemical reaction
8	G_{2}	$1-(1-\alpha)^{3}$	$1/[3(1-\alpha)^{2}]$	Chemical reaction
9	G_{3}	$1-(1-\alpha)^{4}$	$1/[4(1-\alpha)^{3}]$	Chemical reaction
2. Acceleratory rate equations				
10	$P_{3/2}$	$\alpha^{3/2}$	$(2/3)\alpha^{-1/2}$	Nucleation (power law)
11	$P_{1/2}$	$\alpha^{1/2}$	$2\alpha^{1/2}$	Nucleation (power law)
12	$P_{1/3}$	$\alpha^{1/3}$	$3\alpha^{2/3}$	Nucleation (power law)
13	$P_{1/4}$	$\alpha^{1/4}$	$4\alpha^{3/4}$	Nucleation (power law)
14	P_{2}	α^{2}	$(1/2)\alpha^{-1}$	Nucleation (parabolic law)
15	E_{1}	$\ln\alpha$	α	Nucleation (exponential law)
16	E_{2}	$\ln\alpha^{2}$	$\alpha/2$	Nucleation (exponential law)
3. Sigmoidal rate equations or random nucleation and subsequent growth				
17	A_{1}, F_{1}	$-\ln(1-\alpha)$	$1-\alpha$	Random nucleation/first order (Mampel)
18	$A_{2/3}$	$[-\ln(1-\alpha)]^{3/2}$	$(2/3)(1-\alpha)[-\ln(1-\alpha)]^{-1/2}$	Random nucleation (Avrami-Erofeev)
19	$A_{3/2}$	$[-\ln(1-\alpha)]^{2/3}$	$(3/2(1-\alpha)[-\ln(1-\alpha)]^{1/3}$	Random nucleation (Avrami-Erofeev)

No.	Model	*g(α)*	*f(α)*	Rate-determining mechanism
20	$A_{3/4}$	$[-\ln(1-\alpha)]^{4/3}$	$(3/4)(1-\alpha)[-\ln(1-\alpha)]^{-1/3}$	Random nucleation (Avrami-Erofeev)
21	$A_{5/2}$	$[-\ln(1-\alpha)]^{2/5}$	$(5/2)(1-\alpha)[-\ln(1-\alpha)]^{3/5}$	Random nucleation (Avrami-Erofeev)
22	A_{2}	$[-\ln(1-\alpha)]^{1/2}$	$2(1-\alpha)[-\ln(1-\alpha)]^{1/2}$	Random nucleation (Avrami-Erofeev)
23	A_{3}	$[-\ln(1-\alpha)]^{1/3}$	$3(1-\alpha)[-\ln(1-\alpha)]^{2/3}$	Random nucleation (Avrami-Erofeev)
24	A_{4}	$[-\ln(1-\alpha)]^{1/4}$	$4(1-\alpha)[-\ln(1-\alpha)]^{3/4}$	Random nucleation (Avrami-Erofeev)
25	$A_{1/2}$	$[-\ln(1-\alpha)]^{2}$	$1/2(1-\alpha)[-\ln(1-\alpha)]^{-1}$	Random nucleation (Avrami-Erofeev)
26	$A_{1/3}$	$[-\ln(1-\alpha)]^{3}$	$1/3(1-\alpha)[-\ln(1-\alpha)]^{-2}$	Random nucleation (Avrami-Erofeev)
27	$A_{1/4}$	$[-\ln(1-\alpha)]^{4}$	$1/4(1-\alpha)[-\ln(1-\alpha)]^{-3}$	Random nucleation (Avrami-Erofeev)
28	B_{1}	$\ln[\alpha/(1-\alpha)]$	$\alpha/(1-\alpha)$	Branching nuclei (Prout–Tompkins)
4. Deceleratory rate equations (phase boundary reaction)				
29	R_{1}, F_{0}, P_{1}	α	1	Contracting disk
30	R_{2}, $F_{1/2}$	$1-(1-\alpha)^{1/2}$	$2(1-\alpha)^{1/2}$	Contracting cylinder
31	R_{3}, $F_{2/3}$	$1-(1-\alpha)^{1/3}$	$3(1-\alpha)^{2/3}$	Contracting sphere
5. Deceleratory rate equations (equations based on the diffusion mechanism)				
32	D_{1}	α^{2}	$1/(2\alpha)$	One-dimensional diffusion
33	D_{2}	$\alpha+(1-\alpha)\ln(1-\alpha)$	$[-\ln(1-\alpha)]^{-1}$	Three-dimensional diffusion
34	D_{3}	$[1-(1-\alpha)^{1/3}]^{2}$	$(3/2)(1-\alpha)^{2/3}[1-(1-\alpha)^{1/3}]^{-1}$	Three-dimensional diffusion (Jander)
35	D_{4}	$1-(2/3)\alpha-(1-\alpha)^{2/3}$	$(3/2)[(1-\alpha)^{-1/3}-1]^{-1}$	Three-dimensional diffusion (Ginstling–Brounshtein)

No.	Model	$g(\alpha)$	$f(\alpha)$	Rate-determining mechanism
36	D_5	$[(1-\alpha)^{-1/3}-1]^2$	$(3/2)(1-\alpha)^{4/3}[(1-\alpha)^{-1/3}-1]^{-1}$	Three-dimensional diffusion (Crank)
37	D_6	$[(1+\alpha)^{1/3}-1]^2$	$(3/2)(1+\alpha)^{2/3}[(1+\alpha)^{1/3}-1]^{-1}$	Three-dimensional diffusion
38	D_7	$1+(2/3)\alpha-(1+\alpha)^{2/3}$	$3/2[(1+\alpha)^{-1/3}-1]^{-1}$	Three-dimensional diffusion
39	D_8	$[(1+\alpha)^{-1/3}-1]^2$	$3/2(1+\alpha)^{4/3}[(1+\alpha)^{-1/3}-1]^{-1}$	Three-dimensional diffusion
6. Other kinetic equations with unjustified mechanism				
40	G_7	$[1-(1-\alpha)^{1/2}]^{1/2}$	$4\{(1-\alpha)[1-(1-\alpha)^{1/2}]\}^{1/2}$	
41	G_8	$[1-(1-\alpha)^{1/3}]^{1/2}$	$6(1-\alpha)^{2/3}[1-(1-\alpha)^{1/3}]^{1/2}$	

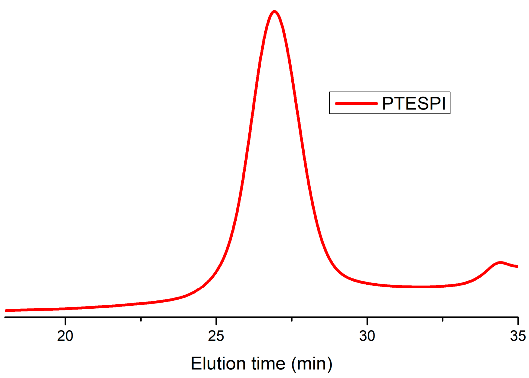

Figure S1: SEC trace of PTESPI in THF

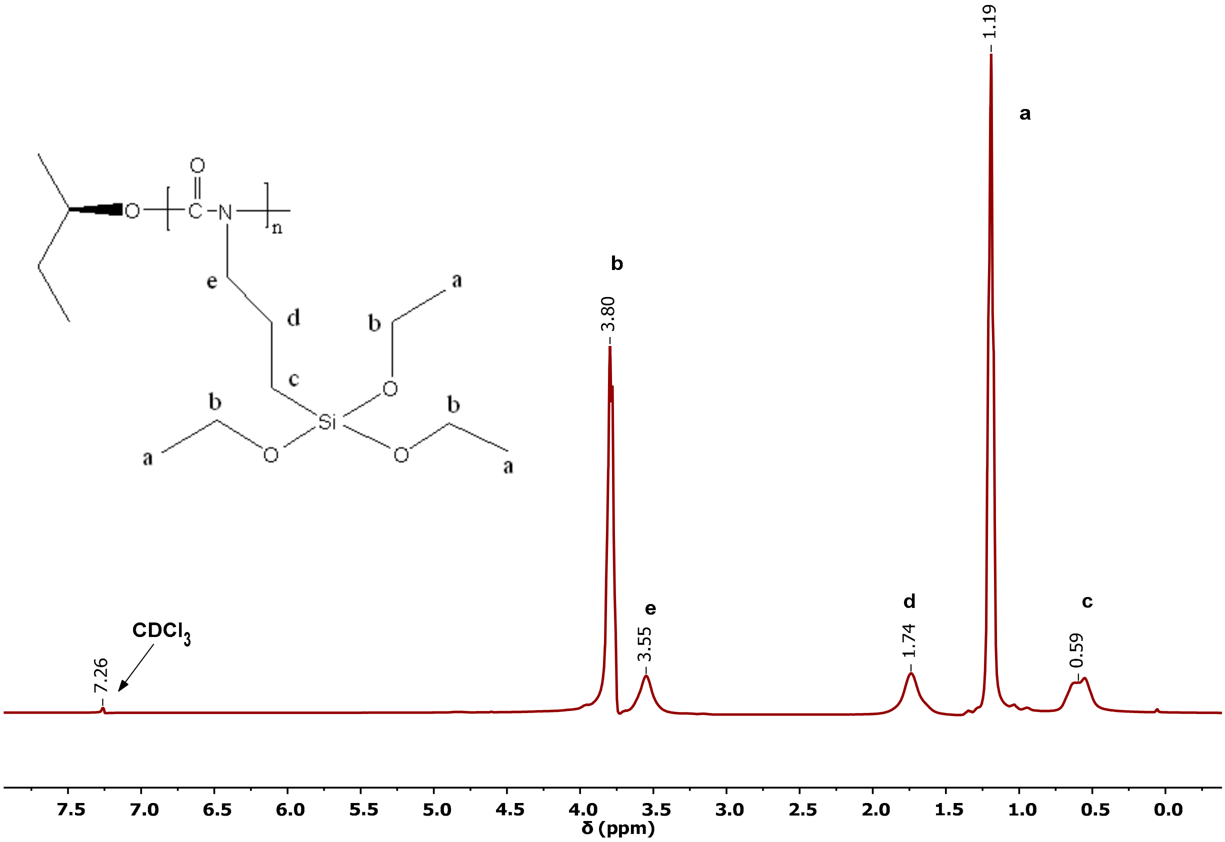

Figure S2: 400 MHz ^{1}H NMR spectrum of PTESPI in $CDCl_3$

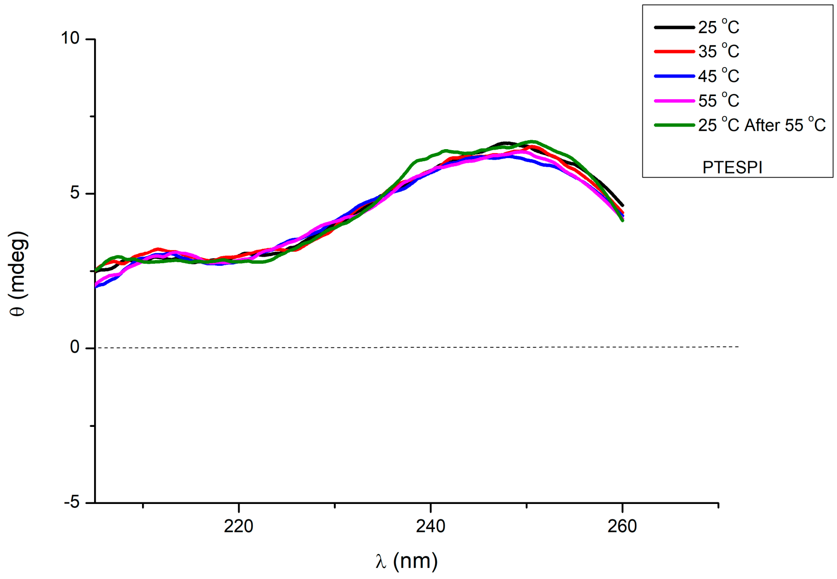

Figure S3: CD spectrum of PTESPI in hexane for various temperatures

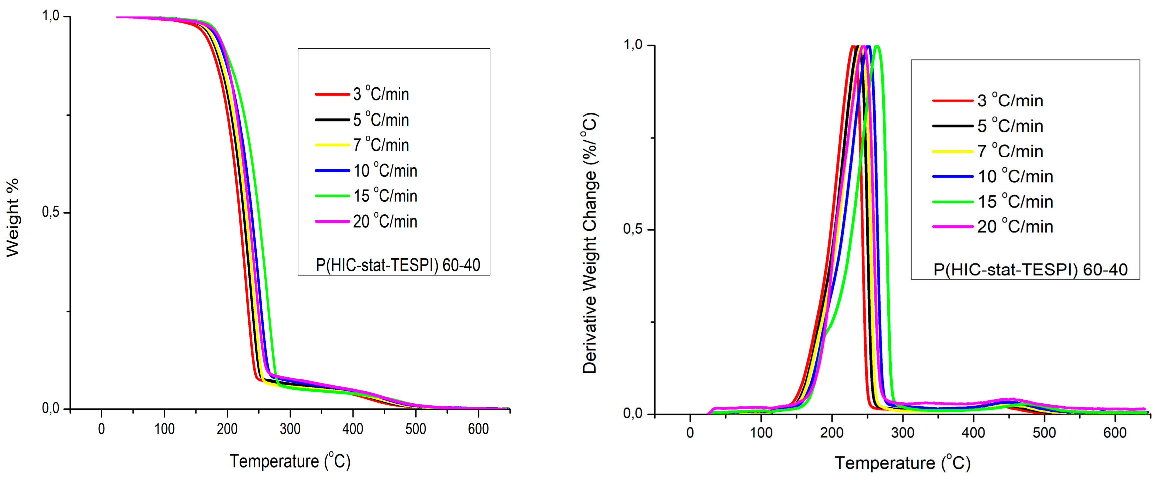

Figure S4: TGA and DTG plots for the statistical copolymer 60/40 at various heating rates

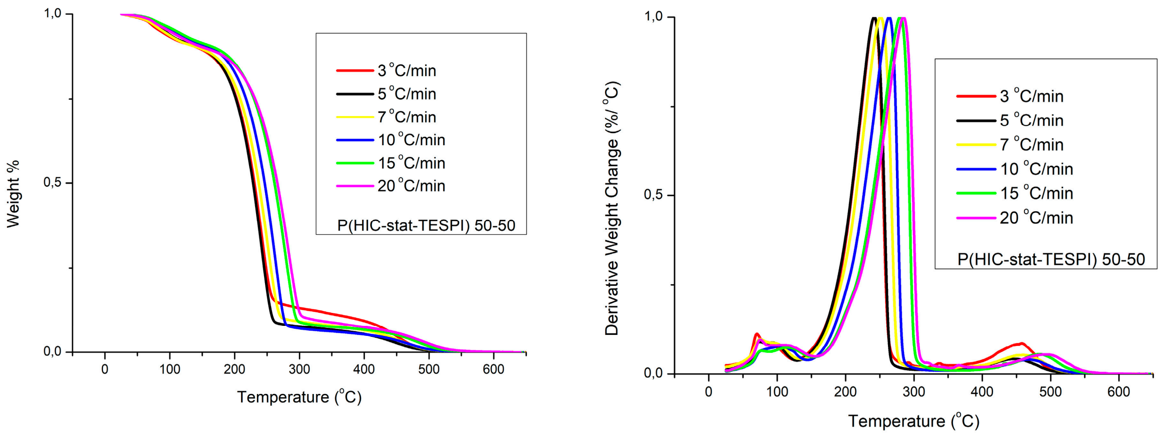

Figure S5: TGA and DTG plots for the statistical copolymer 50/50 at various heating rates

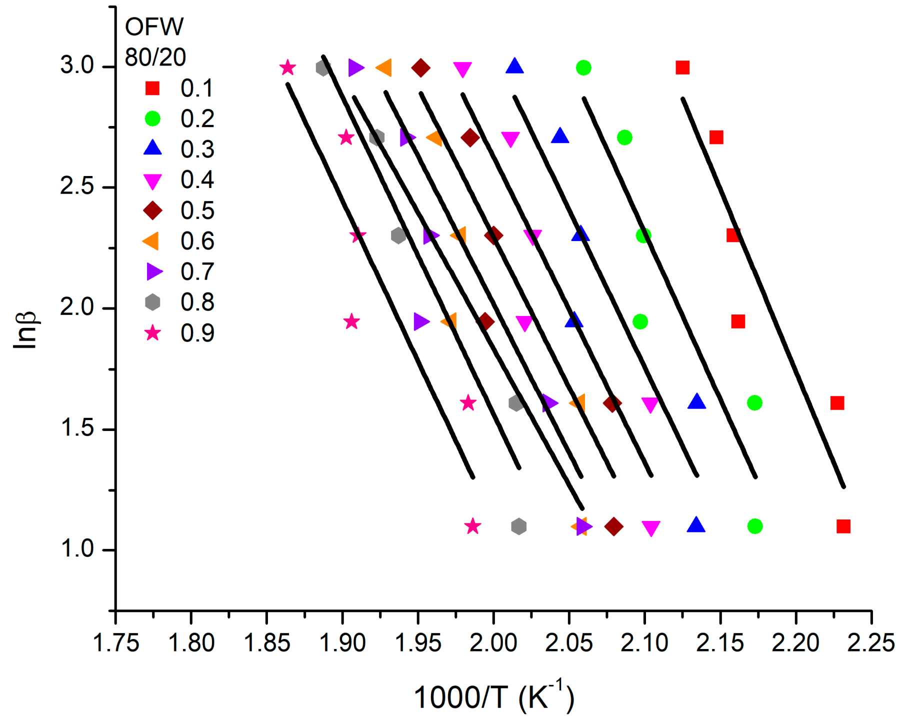

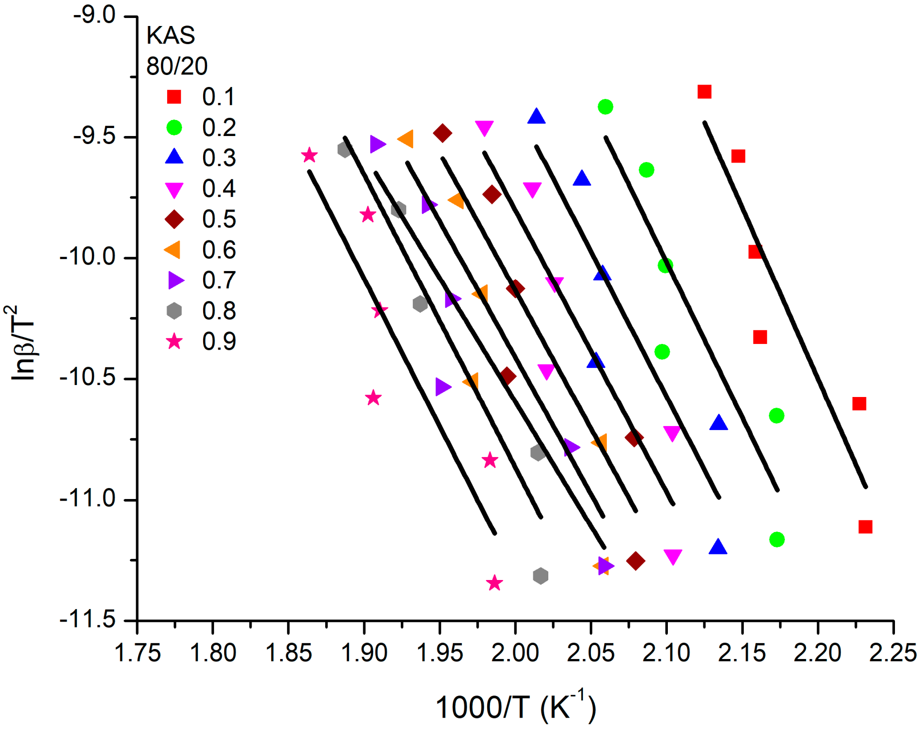

Figure S6: (OFW) and (KAS) plots for the statistical copolymer 80/20

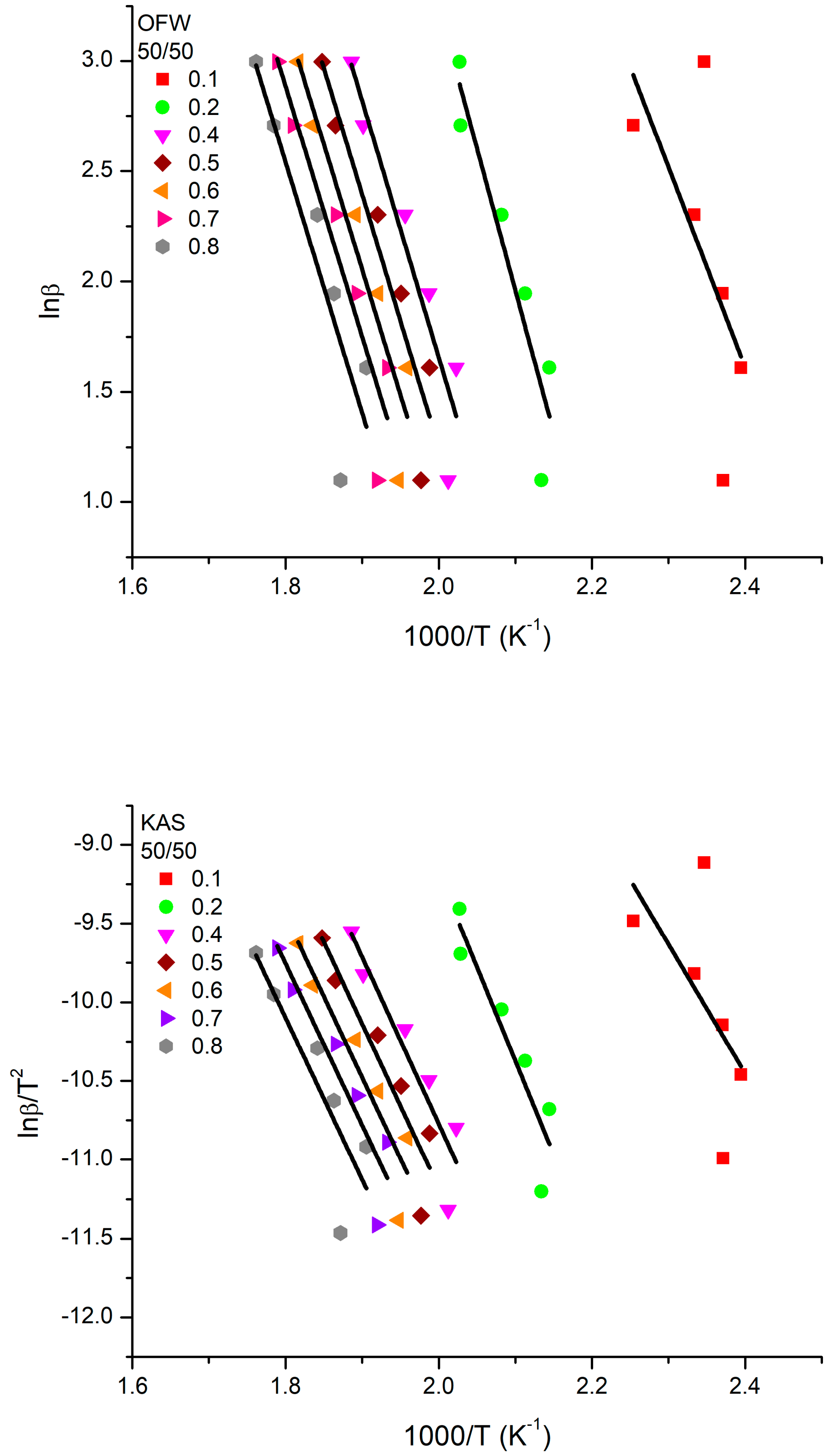

Figure S7: (OFW) and (KAS) plots for the statistical copolymer 50/50

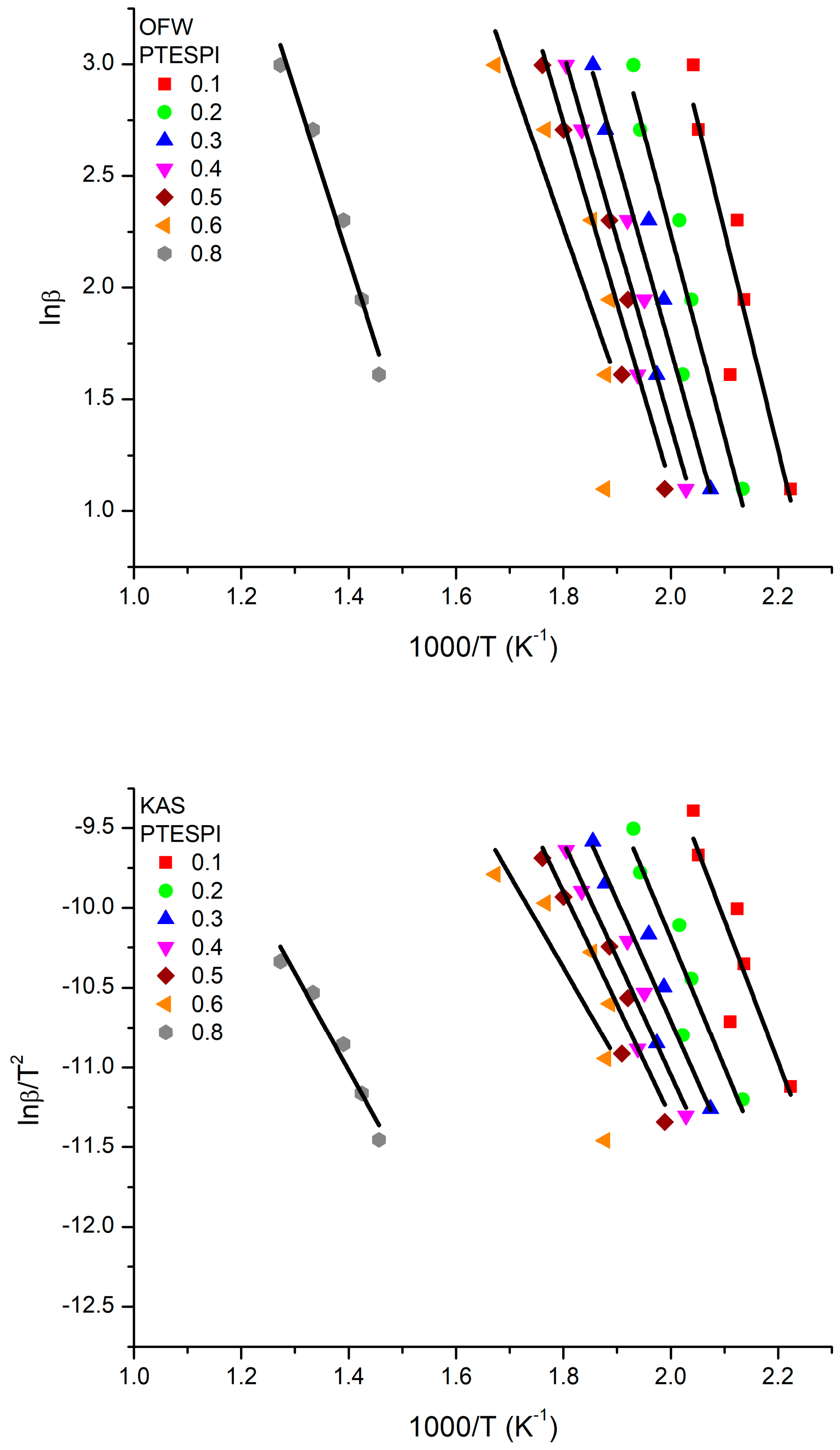

Figure S8: (OFW) and (KAS) plots for PTESPI

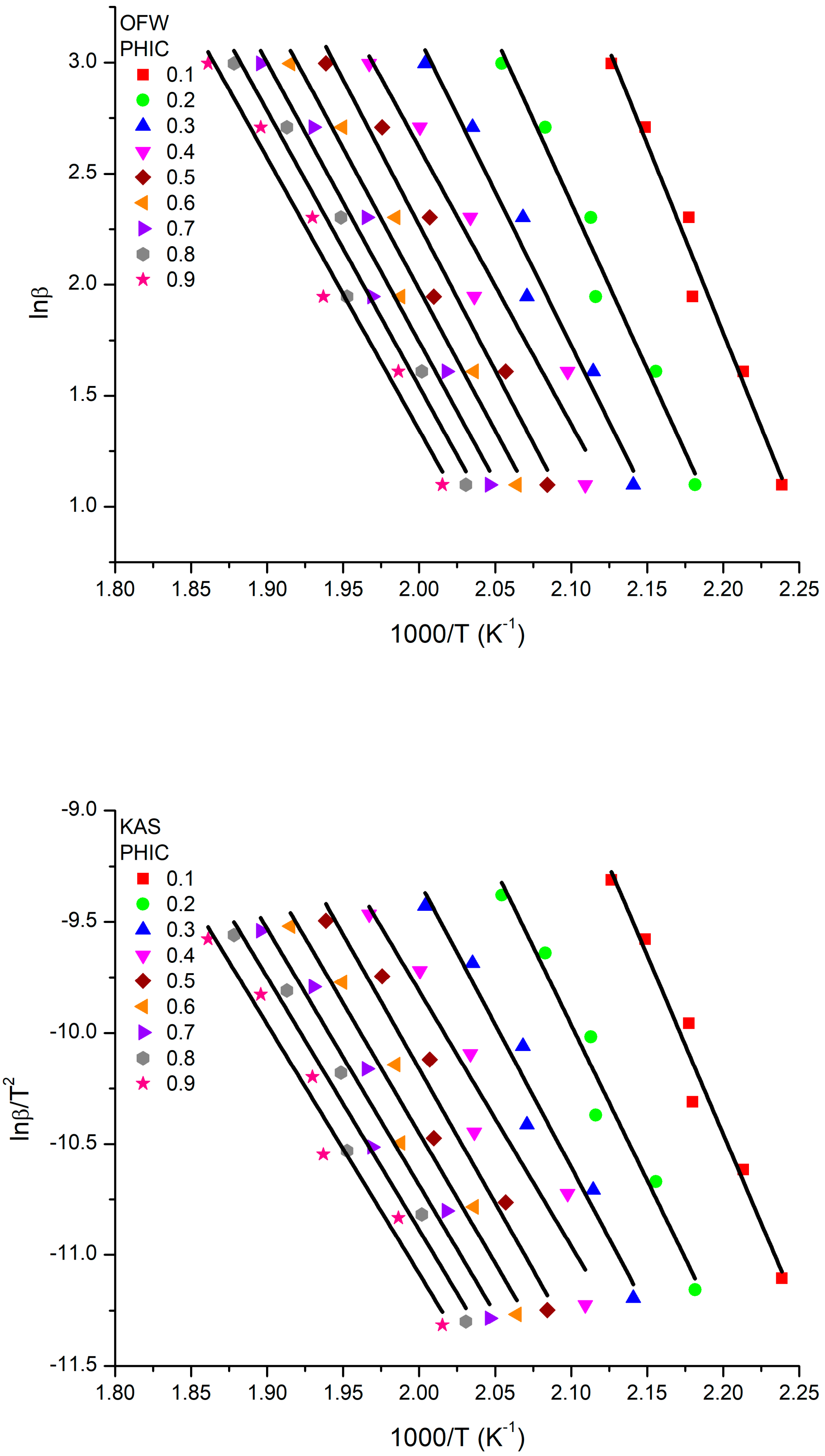

Figure S9: (OFW) and (KAS) plots for PHIC
